# Pattern of Reduced Functional Connectivity and Structural Abnormalities in Parkinson’s Disease: An Exploratory Study

**DOI:** 10.3389/fneur.2016.00243

**Published:** 2017-01-13

**Authors:** Rachel Paes Guimarães, Maria Cristina Arci Santos, Alain Dagher, Lidiane Soares Campos, Paula Azevedo, Luiza Gonzaga Piovesana, Brunno Machado De Campos, Kevin Larcher, Yashar Zeighami, Augusto C. Scarparo Amato-Filho, Fernando Cendes, Anelyssa Cysne Frota D’Abreu

**Affiliations:** ^1^Department of Neurology, University of Campinas, Campinas, Brazil; ^2^Laboratory of Neuroimaging, University of Campinas, Campinas, Brazil; ^3^Montreal Neurological Institute, Brain Imaging Center, McGill University, Montreal, QC, Canada; ^4^Department of Radiology, University of Campinas, Campinas, Brazil

**Keywords:** Parkinson’s disease, neuroimaging, cortical thickness, functional MRI, voxel-based morphometry

## Abstract

**Background:**

MRI brain changes in Parkinson’s disease (PD) are controversial.

**Objectives:**

We aimed to describe structural and functional changes in PD.

**Methods:**

Sixty-six patients with PD (57.94 ± 10.25 years) diagnosed according to the UK Brain Bank criteria were included. We performed a whole brain analysis using voxel-based morphometry (VBM–SPM 8 software), cortical thickness (CT) using CIVET, and resting-state fMRI using the Neuroimaging Analysis Kit software to compare patients and controls. For VBM and CT we classified subjects into three groups according to disease severity: mild PD [Hoehn and Yahr scale (HY) 1–1.5], moderate PD (HY 2–2.5), and severe PD (HY 3–5).

**Results:**

We observed gray matter atrophy in the insula and inferior frontal gyrus in the moderate PD and in the insula, frontal gyrus, putamen, cingulated, and paracingulate gyri in the severe groups. In the CT analysis, in mild PD, cortical thinning was restricted to the superior temporal gyrus, gyrus rectus, and olfactory cortex; in the moderate group, the postcentral gyrus, supplementary motor area, and inferior frontal gyrus were also affected; in the severe PD, areas such as the precentral and postentral gyrus, temporal pole, fusiform, and occipital gyrus had reduced cortical thinning. We observed altered connectivity at the default mode, visual, sensorimotor, and cerebellar networks.

**Conclusion:**

Subjects with mild symptoms already have cortical involvement; however, further cerebral involvement seems to follow Braak’s proposed mechanism. Similar regions are affected both structurally and functionally. We believe the combination of different MRI techniques may be useful in evaluating progressive brain involvement and they may eventually be used as surrogate markers of disease progression.

## Highlights

- Subjects with mild symptoms already have cortical involvement; however, further cerebral involvement seems to follow Braak’s proposed mechanism.
- Similar brain regions are affected both structurally and functionally, such as temporal, frontal, and occipital gyri; however, it is still unclear if functional alterations are consequence or direct cause of structural ones or if they occur simultaneously.

## Introduction

Parkinson’s disease (PD) is a progressive neurodegenerative disorder (1). Considering the variability of symptoms and the still unknown etiology, MRI-based studies are important to better understand disease physiopathology.

Voxel-based morphometry (VBM) (2) assesses gray matter (GM) density or volume, voxel by voxel. A meta-analysis revealed GM atrophy in the left inferior frontal gyrus, left superior temporal gyrus and left insula in patients with idiopathic PD (3). Only two studies evaluated disease progression as measured by the Hoehn and Yahr scale (HY), and their outcomes differed. One revealed minimal statistically significant GM reduction (4), while the other described atrophy in olfactory-related regions (5).

Cortical thickness (CT) analysis is needed to assess cortical surface properties (6) for it allows subvoxel precision, as thickness values are assigned to individual vertices (7). CT is useful to investigate subtle cortical changes in the brain (8). Previous studies using CT in PD demonstrated widespread alterations (9–11), nonetheless a clear pattern was not established.

Resting-state functional MRI (rs-fMRI) evaluates regional and neuronal circuitry at rest (12), as it measures functional connectivity (FC) in spatially distinctive regions (13). rs-fMRI studies in PD found altered connectivity in the cerebellum, primary motor cortex, supplementary motor area (SMA), dorsolateral and prefrontal cortex, and putamen (4).

Wu et al., using FC global measure through graphical analysis, found increased coupling in the cortex and cerebellum and primary motor cortex, and decreased FC in SMA, dorsolateral and prefrontal cortex, and putamen. It is noteworthy that these results are consistent with the altered pattern of metabolic brain activity found in PD using positron emission tomography (14). Decreased FC in a circuit connecting posterior putamen with the inferior parietal cortex as well as an increased FC between subthalamic nuclei and cortical motor areas were also described, indicating abnormalities in sensorimotor integration and suggesting that some symptoms, such as tremor, may be related to an abnormal coupling of these areas (15, 16).

Alterations in CT have also been demonstrated in PD patients in multiple brain regions, such as occipital, temporal, and frontal cortices (10, 17). Pereira et al. demonstrated a widespread cortical thinning in lateral occipital, parietal and temporal, frontal and premotor regions.

Cortical thickness measure is a valuable tool for it allows subvoxel precision because thickness values are assigned to individual vertices instead of voxels (7, 9).

Despite the progress obtained in the last 30 years trying to comprehend PD pathophysiology, several questions remain.

In general, neuroimaging studies in PD compare patients with or without certain disorders, such as dementia and depression, but do not consider associated clinical markers like motor symptoms severity, other non-motor symptoms, or the presence of motor complications (10, 18).

Furthermore, despite the large number of validated scales for clinical evaluation in PD patients, there are few studies correlating them with MRI findings.

One of the greatest challenges nowadays in PD is the absence of biomarkers for disease diagnosis in premotor stages and differential diagnosis with other neurodegenerative causes of parkinsonism. Thus, our purpose was to systematically evaluate patients with PD from clinical and neuroimaging data using rs-fMRI and CT analyzes. However, our focus was to generate hypotheses based in this preliminary study, and a longitudinal study is already under way in order to confirm the findings in this paper.

## Materials and Methods

Cross-sectional study was conducted at the Neuroimaging Laboratory—Unicamp University Hospital and McConnel Brain Imaging Center—Montreal Neurological Institute (MNI) (McGill University). The State University of Campinas Ethics Committee approved the study, and all individuals signed an informed written consent prior to any research related procedure. All images were acquired at UNICAMP University Hospital and MRI analysis was performed at MNI.

### Subjects

Sixty-six patients (57.94 ± 10.25 years) (43 men) older than 30 years with PD, diagnosed according to the UK Parkinson’s Disease Society Brain Bank criteria (19) were consecutively recruited from the Movement Disorders Outpatient Clinic of the University Hospital (UNICAMP), Brazil, and underwent MRI (Table 1). In 46, we performed the whole clinical protocol, including the clinical scales. An experienced neurologist specialized in Movement Disorders assessed all patients.

**Table 1:** **Demographic data from all Parkinson’s disease (PD) patients, mild PD, moderate PD, and severe PD**.

	All PD		Mild PD		Moderate PD		Severe PD	
Clinical data	Mean	SD	Mean	SD	Mean	SD	Mean	SD
Age	59.33	9.8	59.31	9.60	58.5	10.69	62.07	8.4
Education	6.9	4.7	8.05	4.30	7.05	5.01	4.84	3.3
Unified Parkinson’s Disease Rating Scale (UPDRS)	35.74	18.47	20.94	8.86	34	13.38	63.21	17.15
UPDRS-III	16.93	8.23	10.88	78	16.2	5.95	28.35	8.88
Hoehn and Yahr scale	2.8	1.26	1.25	0.25	2.57	0.3	4.42	0.51
Scales for Outcomes in Parkinson’s Disease—Cognition (SCOPA-COG)	19.24	6.8	21.66	3.77	19.69	7.18	13.28	7.15
SCOPA-CP	3.6	7.8	1.55	1.72	2.77	2.37	10.07	18.63
NMSS	69.57	48.19	42.72	34.97	71.61	46.95	96.53	52.43
SCHWAB (%)	73	22	88	8	76	15	34	22
Time of disease	7	6.43	2.5	4.08	7	6.62	12.14	5.31
Medication	5.4	5.7	2.5	1.9	3	2.6	9.5	6.8

Evaluation consisted of a standardized questionnaire regarding sex, age, age at disease onset, family history, professional history, environmental exposure to risk factors, and medication used, as well as the application of the Unified Parkinson Disease Rating Scale (UPDRS), HY, Scales for Outcomes in Parkinson’s Disease—Cognition (SCOPA-COG), Schwab and England Activities of Daily Living, and Non-Motor Symptom Assessment Scale (NMSS) (Table 1). All patients were on medication on clinical assessment and the MRI acquisition.

We also included 40 healthy controls (HC), with no history of neurological disorders, no family history of PD, and a normal neurological examination (mean age 57.60 ± 10.77).

For VBM and CT, we classified subjects into three groups according to disease severity: mild PD (HY 1–1.5), moderate PD (HY 2–2.5), and severe PD (HY 3–5) (Table 1).

We performed VBM analysis in 66 patients and 40 HC. Eight patients were excluded from the CT analysis due to excessive head movement or the presence of artifacts. The fMRI acquisition protocol was included after the initial recruitment, so only 48 patients and 33 HC had available images.

### MRI Acquisition

Patients and HC underwent the same MRI acquisition protocol on a 3-T Philips Achieva; MRI scanner at UNICAMP:

(1) Echo-planar imaging sequences of 6 min with isotropic voxel of 3 mm × 3 mm × 3 mm, no gap, 39 slices, FOV = 240 mm × 240 mm, TE = 30 ms, TR = 2000 ms, flip angle = 90°, and 180 volumes (dynamics), in a 6-min scan;
(2) Volumetric (3D) T1 WI, acquired in the sagittal plane with isotropic voxel with 1mm^3^, no gap, flip angle = 35°, TR = 7.1 ms, TE = 3.2 ms, FOV = 240 mm × 240 mm, in a 6-min scan.

### VBM Analysis

We used the VBM8 toolbox of the statistical parametric mapping (SPM8)^1^ and the Diffeomorphic Anatomical Registration Exponentiated Lie Algebra (Dartel) software on Matlab R2012b platform to process and analyze the images. VBM allows a voxel-wise comparison of local GM differences between two groups. VBM procedure involves the segmentation of the original structural MRI images in native space in GM, white matter (WM), and cerebrospinal fluid (CSF) tissues, followed by GM and WM images normalization to templates in stereotactic space to acquire optimized normalization parameters, which are applied to the raw images. GM images were smoothed using an 8-mm full width at half maximum (FWHM) isotropic Gaussian kernel. Finally, we employed a general linear model (GLM), using age and sex as covariates. We obtained the results showing regions of GM concentration with significant differences between the groups (20). Correction for multiple comparisons used the Random Fields Theory (RFT). In each group analysis, a homogeneity test using covariance led to the exclusion of: five patients (PD versus HC); one patient and one HC (mild PD versus HC); three patients and one HC (moderate PD versus HC); and five patients and one HC (severe PD versus HC). The results were displayed using SPM8 and xjView (Human Neuroimaging Lab, Baylor College of Medicine, Houston, TX, USA). We assessed group comparisons by SPM using the family wise error (FWE) at a threshold of *p* < 0.05, covariates were sex and age, with an extent threshold of *K* = 20 voxels and clusters >30 voxels. Brain areas were localized according to the automated anatomical labeling available on The Online Brain Atlas Reconciliation Tool (21).

### CT Analysis

Excessive head movement or the presence of artifacts led to the exclusion of subjects. We used the fully automated pipeline CIVET (version 1.1.10; MNI at McGill University, Montreal, QC, Canada) to estimate CT. Since the fMRI acquisition protocol was also included after the initial recruitment, 48 patients and 33 HC had available images.

In summary, it consists of: correction for magnetic inhomogeneity, skull stripping, linear, and non-linear registration of the native image to the symmetric ICBM 152 template; tissue classification into GM, WM, and CSF, corrected for the partial volume effect; GM and WM surfaces are extracted using the Laplacian map; thickness is calculated at each vertex using the *t*- link metric method. CT is estimated as the distance, in millimeters, between WM and GM surfaces at each vertex. More details on this technique can be found on the CIVET website.^2^ For this analysis, we regressed out age. We also correlated scales scores with CT from all PD patients.

After that, we stratified patients into mild (*n* = 16), moderate (*n* = 21), and severe PD (*n* = 11) and compared them to HC. Statistical analysis was performed in Matlab (R2008b, The Mathworks, Natick, MA, USA), using the SurfStat toolbox.^3^ We performed a GLM to describe CT as a combination of demographic variables such as age, group (PD or HC), and sex. We used *p* < 0.05 (RTF).

### rs-fMRI Analysis

The fMRI data were preprocessed using the Neuroimaging Analysis Kit (NIAK) release 0.7 (22).^4^ The first three volumes of each run were omitted to allow the magnetization to reach equilibrium. Each fMRI dataset was corrected for inter-slice differences in acquisition time and the parameters of a rigid-body motion were estimated for each time frame. Rigid-body motion was then estimated between runs. The median volume of the first run for each subject was co-registered with a T1 individual scan (23), which was itself non- linearly transformed to MNI space, using the latest version of the ICBM152 template (24). The transformations fMRI-to-T1 and T1-to-stereotaxic were concatenated, to resample the functional volumes into MNI space at a 3-mm isotropic resolution. The “scrubbing” method of Power et al. (25), was used to remove volumes with excessive motion. The fMRI volumes were spatially smoothed with a 6-mm isotropic Gaussian blurring kernel. A more detailed description of the pipeline can be found at: https://github.com/ulrikls/niak. We performed a Bootstrap analysis of stable clusters (BASC). It consists in studying the stability of networks detected with iterative operations of clustering. The resulting stability matrix is used in a clustering procedure, which derives so-called stable clusters. BASC is first applied on individual fMRI time series, and applying a Circular Block Bootstrap to fMRI time series derives replications of clustering. A stability matrix is derived for each subject. The average individual stability matrix is then computed and a hierarchical clustering is applied on it. This allows defining group-level clusters that maximize the average probability of being clustered at the individual level. By bootstrapping the subject stability matrices, the group-level stability matrix is derived. A hierarchical clustering is finally applied on the group stability matrix to define stable group clusters. For each brain region, the average stability of that region with every other region in the cluster of the seed can be derived.

We excluded patients with excessive head movement, since it could result in spurious observations; however, little head movement was accepted (frame displacement lower than 0.2).

Statistical analysis was performed in Matlab (R2008b, The Mathworks, Natick, MA, USA). We performed a GLM to describe connectivity as a combination of variables such as age, group (PD or HC), sex, with head motion as a covariate. We selected a *p* < 0.05.

### Statistical Analysis

For statistical analysis, we used STATA 13.1 version. We compared baseline demographic characteristic using a one-way Analysis of Variance, without covariates, followed by a *post hoc* Sidak test. For categorical variables, we performed a chi-square test. For correlation analyses, we perform a GLM, including the scales scores as independent variables and sex and age as covariates. Level of significance was established at *p* < 0.05.

## Results

### Voxel-Based Morphometry

**Figure 1:**
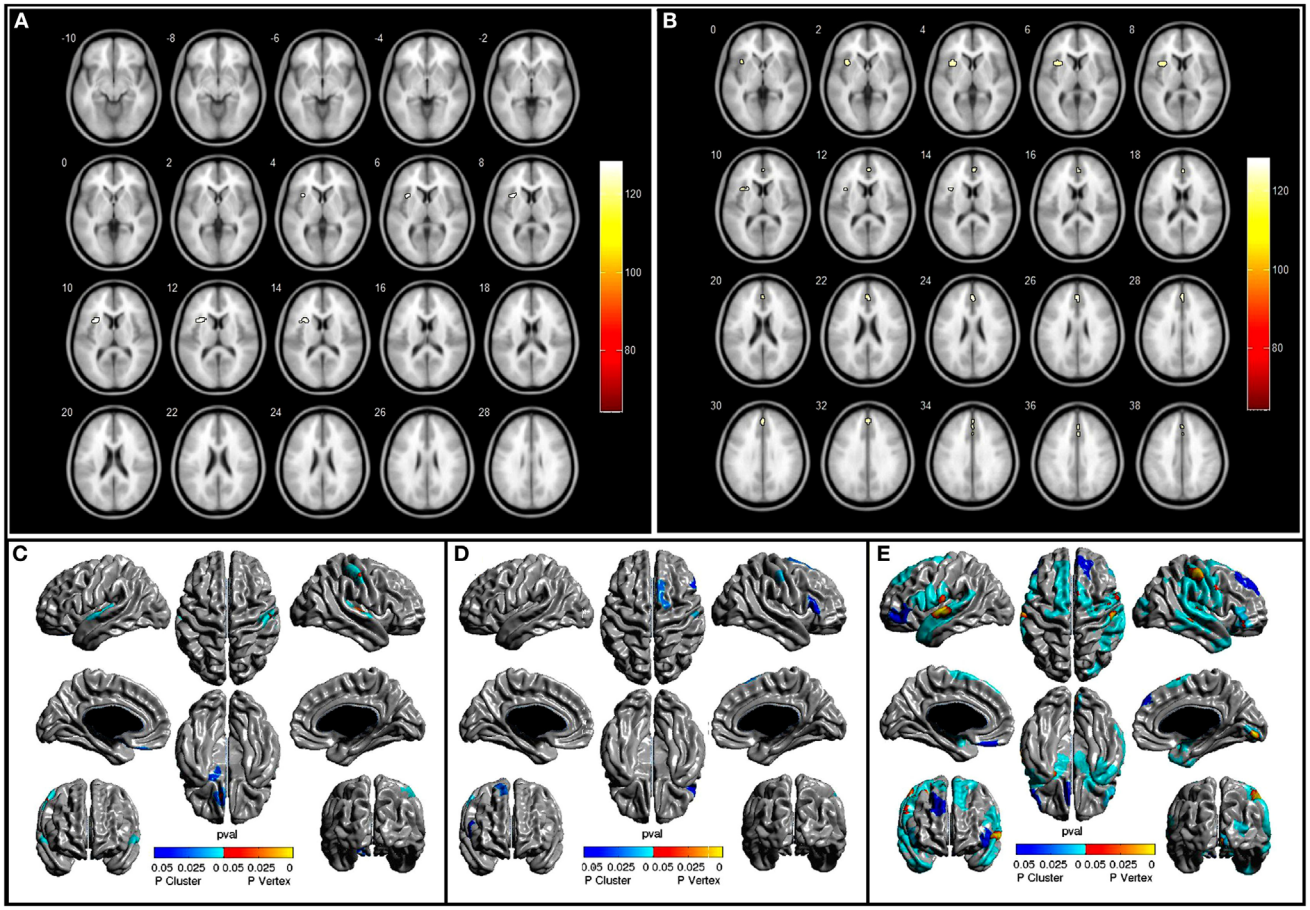
**(A,B)** show areas with atrophy in moderate PD and severe PD patients when compared to healthy controls (HC), *p* < 0.05 FEW, **(C–E)** show area with cortical thinking in mild PD, moderate PD, and severe PD, respectively, when compared to HC *p* < 0.5, Random Fields Theory.

Mild PD (*n* = 14) versus HC: there was no GM reduction in any region (FWE *p* < 0.05) (Figure 1; Table 2). Moderate PD (*n* = 31) versus HC: VBM detected GM loss in the left insula and left inferior frontal gyrus, opercular part (FWE *p* < 0.05). Severe PD (*n* = 16) versus HC: we observed GM atrophy in the left insula, left inferior frontal gyrus (opercular part), left putamen, left and right medial superior frontal gyrus, left and right anterior cingulate and paracingulate gyri, and right median cingulated, and paracingulate gyri (FWE *p* < 0.05).

**Table 2:** **Areas with reduced cortical thickness (CT) on the left and GM atrophy on the right of mild Parkinson’s disease (PD) versus healthy controls (HC), moderate PD versus HC and severe PD versus HC (*p* < 0.05)**.

CT			Voxel-based morphometry		
Coordinates (*x y z*)	Areas	*p* [random fields Theory (RFT)]	Coordinates (*x y z*)	Areas	*p* (RFT)
**Mild PD**					
−48 −11 0	L superior Temporal Gyrus	<0.05	–	–	–
−9 31 −21	Gyrus rectus	<0.05	–	–	–
−21 9 −18	L superior frontal gyrus	<0.05	–	–	–
−16 6 −16	L olfactory cortex	<0.05	–	–	–
55 −16 46	R post central gyrus	<0.05	–	–	–
**Moderate PD**					
14 1 69	Supplementary motor area (SMA)	<0.05	−30 14 9	L insula	<0.05
53 25 9	Inferior frontal gyrus	<0.05	−30 14 9	L inferior frontal gyrus	<0.05
16 0 71	R superior frontal Gyrus	<0.05			
**Severe PD**					
−41 21 9	L inferior frontal gyrus	<0.05	−35 14 3	L insula	<0.05
−64 -12 17	L postcentral gyrus	<0.05	−35 14 3	L inferior frontal gyrus	<0.05
35 −10 −38	Fusiform gyrus	<0.05	−35 14 3	L putamen	<0.05
−29 −15 70	L precentral gyrus	<0.05	0 41 25	R superior frontal gyrus	<0.05
−9 −1 70	L SMA	<0.05	0 41 25	Anterior cingulum	<0.05
−14 −12 67	L superior frontal gyrus	<0.05	0 33 36	L superior frontal gyrus	<0.05
−7 −11 71	Paracentral lobule	<0.05	–	–	–
51 4- 34	R medial temporal gyrus	<0.05	–	–	–
25 14 −40	R superior temporal gyrus	<0.05	–	–	–

### Cortical Thickness

Mild PD (*n* = 16) versus HC: CT analysis revealed cortical thinning in the left superior temporal gyrus, left gyrus rectus, and left olfactory cortex (RFT *p* < 0.05). Moderate PD (*n* = 21) versus HC: the areas with cortical thinning were the right postcentral gyrus, right SMA, and right inferior frontal gyrus (triangular and opercular parts) (RFT *p* < 0.05) (Figure 1; Table 2). Severe PD (*n* = 11) versus HC: CT analysis revealed cortical thinning in the left inferior frontal gyrus, left precentral and postcentral gyrus, left SMA, left inferior frontal gyrus (triangular part), left gyrus rectus, right temporal pole, right fusiform gyrus, right middle temporal gyrus, and right occipital gyrus (RFT *p* < 0.05). We found a negative correlation between UPDRS and CT in superior temporal gyrus, pre and post central gyrus, superior and inferior frontal gyrus, SMA, occipital gyrus, and gyrus rectus. The same negative correlation was found regarding UPDRS-III in superior and medial temporal gyrus, lingual gyrus, parahipocampal gyrus, SMA, and medial temporal gyrus (*p* < 0.05). SCOPA and NMSS scores did not correlate with CT values.

### Resting-State Functional MRI

**Figure 2:**
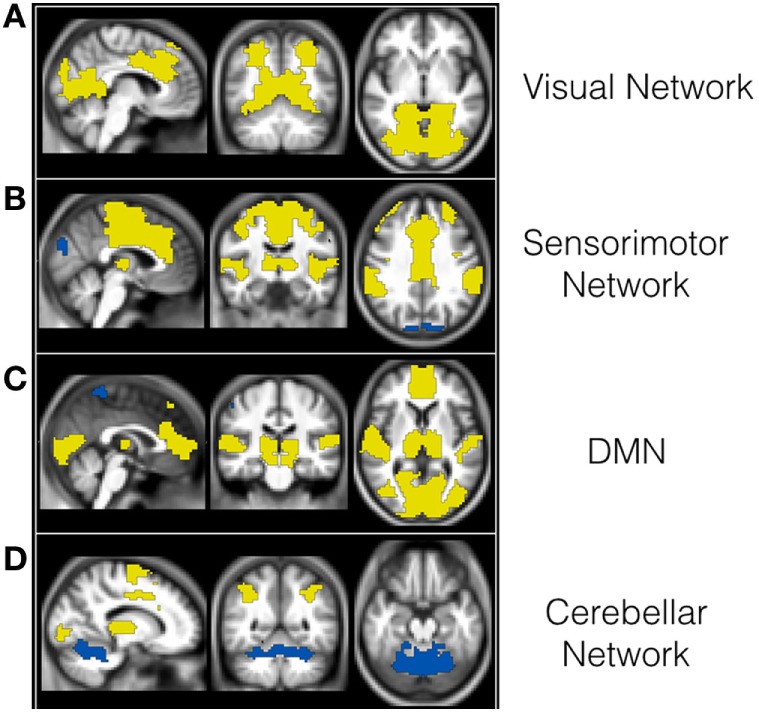
**Reduced functional connectivity (FC) within visual (A), sensorimotor (B), DMN (C), and cerebellum networks (D) in PD patients when compared to controls**. The areas in yellow have reduced FC with areas in blue in PD patients compared to controls at *p* level 0.05.

The final sample was too small to further subdivide, so we performed a whole brain analysis, without an *a priori* assumption (Figure 2; Table 3). Our main objective was to demonstrate by cluster analysis that the resting-state abnormalities occur in the whole brain in PD patients. Based on data provided by the BASC pipeline, we selected the scale with 70 clusters as the most stable one and examined the networks found at this scale (22). We found reduced connectivity within visual, sensorimotor, DMN, and cerebellum networks.

**Table 3:** **Areas with reduced functional connectivity in Parkinson’s disease patients when compared to healthy controls within clusters (*p* < 0.05)**.

Cluster	Coordinate (*x y z*)	Networks	Areas with altered connectivity	*p*-Value
16	−6 58 −2	Visual	Parhippocampal gyrus	<0.05
			Lingual gyrus	<0.05
			Occipital pole	<0.05
			Superior parietal lobule	<0.05
			Precentral gyrus	<0.05
			Supramarginal gyrus	<0.05
38	−3 10 34	Sensorimotor	Thalamus	<0.05
			Insula	<0.05
			Superior temporal gyrus	<0.05
			Precentral gyrus	<0.05
			Inferior frontal gyrus	<0.05
			Middle frontal gyrus	<0.05
			Parietal lobule	<0.05
			Supramarginal gyrus	<0.05
			Cingulate Gyrus	<0.05
66	0 −22 4	DMN	Thalamus	<0.05
			Occipital lobe	<0.05
			Lingual gyrus	<0.05
			Anterior cingulate	<0.05
			Cingulate gyrus	<0.05
			Superior frontal gyrus	<0.05
			Superior temporal gyrus	<0.05
69	0 −67 −14	Cerebellar	Lingual gyrus	<0.05
			Thalamus	<0.05
			Cingulate Gyrus	<0.05
			Precentral gyrus	<0.05
			Frontal gyrus	<0.05
			Angular gyrus	<0.05
			Frontal pole	<0.05
			Occipital cortex	<0.05
			Brainstem	<0.05

## Discussion

We evaluated PD patients using VBM, CT, and rs-fMRI analysis without an *a priori* assumption. GM volume and CT are the two most widely used measures for detecting GM morphometric changes, but they measure different aspects of the brain, hence the importance of performing both methods (8). We observed a certain concurrence between VBM and CT findings within each stage.

One region with decreased CT in mild PD was the left olfactory cortex, which receives sensory information from the olfactory bulb (26), which is in keeping with the olfactory loss as one of the early non-motor manifestations of PD as well as with previous findings of Lewy pathology in anterior olfactory nucleus and olfactory bulb (27). We did not find the same alteration using VBM; however, CT analyses are more sensitive than VBM for identifying regional cortical thinning associated with PD because VBM merges information about morphology, size, and position and the final measures average information about thickness and cortical folding (2), possibly resulting in less sensitivity.

Olfactory loss is an important non-motor manifestation in PD, and it seems to be present years before diagnosis (28). We could not find an association between NMSS scores and CT; however, we used the scale total score, and possibly, an assessment of each scale domain is more appropriate for PD, especially in longitudinal analysis. Previous studies demonstrated a correlation between amygdala and hippocampus volume with depression and anxiety in PD patients (29); however, these symptoms are non-specific and can be present in normal aging (30).

The widely known and accepted Braak’s stage postulates that the Lewy body (LB) deposition in PD progresses in an ascending fashion (31). In stages 1 and 2, it is essentially restricted to the medulla oblongata. In stages 3 and 4, the involvement is mostly observed in the brain stem without cortical lesions (stage 3) or with initial deposition at the anteromedial temporal mesocortex (stage 4), while in stages 5 and 6, there is inexorable cortical involvement. Recent studies suggest the possibility that α-synuclein is a prion-like protein and that PD is a prion-like disease, a theory compatible with Braak’s staging system (32). Other pathological studies, however, have clearly challenged the generalizability of this hypothesis, and as far as we know, it has not been demonstrated by *in vivo* studies (33).

Considering our findings, even patients with mild motor symptoms, already have cortical involvement (possibly stage 4/5), while we did not observe significant atrophy in the brainstem. We believe this is due to a compensatory mechanism of the brain, where brain alterations are already present but there is still no clinical symptoms.

The further progression seems to follow the previously described pattern. It is worth mentioning, nevertheless, that while the Braak’s stage takes LB depositions into consideration, imaging analyses are mostly concerned with GM loss. Hence, some of the differences in the results may reflect the variable under study, and its measurement and not the validity of the findings. VBM is also not a particular sensitive tool for posterior fossa analysis, and the presence of LB does not necessarily imply measurable cortical volume change. Moreover, previous studies suggested that synaptic dysfunction can occur without the presence of Lewy bodies *per se* and that using the presence of LB to measure pathological progression might not be enough to predict the spread of disease (34).

Nevertheless, the importance of these findings is clear. Pathology is far advanced throughout the brain when patients are actually diagnosed with the disease. Even though this has been suggested by the knowledge and identification of non-motor symptoms in the premotor phase (35), it had not been universally demonstrated in idiopathic PD (4). This is a fundamental issue when addressing neuroprotective therapies. Since cortical areas are involved at the onset of motor symptoms, even patients with very early disease may not be the ideal candidates for clinical studies. The process of brain pathology may be so advanced that those therapies may no longer work, or possibly longer periods of observation would be necessary to address neuroprotection. Also, PD has diverse clinical manifestations and possibly different PD subtypes may have different underlying pathological patterns. Future studies may consider studying the posterior fossa in detail in subjects with mild or very early disease, such as the SUIT tool for VBM (36).

Since our study is a cross-sectional analysis of disease stages, we can only infer progression of the cerebral involvement as the clinical manifestations worsens. Previous longitudinal studies using different imaging analysis have suggested that striatal atrophy occurs earlier in the disease process, while cortical GM loss is restricted to later stages, reduced overall gyrification, and bilaterally in the inferior parietal, postcentral, precentral, superior frontal, and supramarginal areas was present in patients with disease for less than 1 year, and the rate loss was accelerated as disease progressed (37). Although our subjects presented some homogeneity in their mean clinical scale scores per group (with lower scores in the mild group, intermediate in the moderate group, and higher scores in the severe group in the motor and non-motor scales), we classified them solely based on their HY score. The HY is a well-accepted scale, as it correlates with imaging studies of dopamine loss and with quality of life, motor impairment, and disability scales (38). However, it does not offer any direct information regarding non-motor issues. It should be also noted that the age in the groups were inadvertently similar. We did not pair the three samples based on age. So, more severe subjects had longer disease duration and an earlier onset. Patients with earlier onset usually have a less aggressive disease, compared to those with a later onset (39). It has been previously described that brain alteration in patients with earlier onset and long disease duration fit the Braak’s model, while in patients with later onset and short disease duration it did not occur (40). Thus, we may have underestimated the degree of atrophy in the severe group and a longitudinal approach, taking age of onset and possibly clinical subtypes is the next step in this exploratory study.

Functional connectivity was decreased within visual, sensorimotor, DMN, and cerebellar networks. Although there is overwhelming evidence that several networks are altered in PD (41–43), the results from previous studies are controversial. The methods used are as diverse as the results. They differ in subject samples, type of MRI analysis, and the ROI chosen, making them difficult to compare. Most studies with fMRI use the independent component analysis (ICA) technique, which is based on the theory that the fMRI signal of each voxel represents a linear mixture of signals. These are separated by statistical analysis of independent signals, and finally, the brain regions with the same independent signals are grouped together as separate components (44). ICA, however, finds functional networks in data randomly generated, since the algorithm used is an optimization procedure, and values can vary due to the chosen threshold and variability among patients (45). Conversely, the BASC method provides a fully automated alternative that applies to an arbitrarily large number of networks (22). BASC renders more robust results since it uses the growth region to find networks and observes the replicability of these through the clusters stability analysis.

Unfortunately, our sample was relatively small and heterogeneous, and we were unable to classify the subjects into the same groups in the rs-fMRI analysis. We can only conclude from our analysis that there is widespread decreased FC in PD, and this correlates with the regions in which we observed atrophy and decreased CT in our structural analysis. Different patterns of reduced connectivity may explain differences in clinical presentation within patients with PD, such as dyskinesias (46) and behavioral differences (47). In the future, we may be able to study the pattern of FC in the individual patient, and from the configuration observed, there is a chance we may be able to predict how the disease will progress, or select individualized targets for neurosurgical procedures.

## Conclusion

In summary, we used validated, complementary, and well-documented techniques to assess brain alterations in PD. We confirmed that subjects with mild symptoms already have cortical involvement and similar regions are affected both structurally and functionally. Nevertheless, future longitudinal studies are needed to further comprehend all the mechanisms contributing to PD and its clinical subtypes.

## Ethics Statement

This study was carried out in accordance with the recommendations of Campinas State University ethics committee with written informed consent from all subjects. All subjects gave written informed consent in accordance with the Declaration of Helsinki. The protocol was approved by the Campinas State University ethics committee.

## Author Contributions

Research project conception: RG, MS, AD, ACD, and FC. Research project organization: RG, MS, ACD, and FC. Research project execution: RG, MS, LC, LP, PA, AA-F, KL, and YZ. Statistical analysis design: RG, MS, AD, BC, KL, and YZ. Statistical analysis execution: RG, MS, BC, and KL. Statistical analysis review and critique: AD, ACD, and FC. Manuscript preparation and writing of the first draft: RG and MS, review and critique: AD, ACD, FC, and AA-F.

## Conflict of Interest Statement

The authors declare that the research was conducted in the absence of any commercial or financial relationships that could be construed as a potential conflict of interest. The reviewer GM and handling Editor declared their shared affiliation, and the handling Editor states that the process nevertheless met the standards of a fair and objective review.

## Footnotes

^1^http://www.fil.ion.ucl.ac.uk/spm.

^2^http://www.bic.mni.mcgill.ca/ServicesSoftware/CIVET.

^3^http://www.math.mcgill.ca/keith/surfstat/.

^4^https://github.com/ulrikls/niak.
